# Ellagitannins from *Castanea sativa* Mill. Leaf Extracts Impair *H. pylori* Viability and Infection-Induced Inflammation in Human Gastric Epithelial Cells

**DOI:** 10.3390/nu15061504

**Published:** 2023-03-21

**Authors:** Stefano Piazza, Giulia Martinelli, Marco Fumagalli, Carola Pozzoli, Nicole Maranta, Flavio Giavarini, Luca Colombo, Giovanna Nicotra, Silvia Francesca Vicentini, Francesca Genova, Emma De Fabiani, Enrico Sangiovanni, Mario Dell’Agli

**Affiliations:** 1 Department of Pharmacological and Biomolecular Sciences “Rodolfo Paoletti”, University of Milan, 20133 Milan, Italy; stefano.piazza@unimi.it (S.P.); giulia.martinelli@unimi.it (G.M.); marco.fumagalli3@unimi.it (M.F.); carola.pozzoli@unimi.it (C.P.); nicole.maranta@unimi.it (N.M.); flavio.giavarini@unimi.it (F.G.); genova.francesca@hsr.it (F.G.); mario.dellagli@unimi.it (M.D.); 2 Consorzio Castanicoltori di Brinzio, Orino e Castello Cabiaglio, Società Cooperativa Agricola-Varese, 21100 Varese, Italy; info@consorziocastanicoltori.it; 3 Estratti Piante Officinali (E.P.O.) S.r.l, 20141 Milan, Italy; gnicotra@eposrl.com (G.N.); svicentini@eposrl.com (S.F.V.)

**Keywords:** *Castanea sativa* Mill., gastritis, chestnut, tannins, ellagitannins, castalagin, *Helicobacter pylori*

## Abstract

*Helicobacter pylori* (*H. pylori*) is an etiologic factor of peptic ulcer disease and gastric cancer. Virulent strains of *H. pylori* are correlated with the severity of gastritis, due to NF-κB activation and IL-8 expression at the epithelial level. Ellagitannins have been documented for antibacterial and anti-inflammatory activities, thus suggesting their potential use in gastritis. Recently, several authors, including our group, demonstrated that tannin-rich extracts from chestnut byproducts, at present considered agricultural waste, display promising biological activities. In this work, we detected high levels of polyphenols in hydroalcoholic extracts from chestnut leaves (*Castanea sativa* L.). Among polyphenols, the ellagitannin isomers castalagin and vescalagin (about 1% *w/w* of dry extract) were identified as potential bioactive compounds. In GES-1 cells infected by *H. pylori*, leaf extract and pure ellagitannins inhibited IL-8 release (IC_50_ ≈ 28 µg/mL and 11 µM, respectively). Mechanistically, the anti-inflammatory activity was partly due to attenuation of NF-κB signaling. Moreover, the extract and pure ellagitannins reduced bacterial growth and cell adhesion. A simulation of the gastric digestion suggested that the bioactivity might be maintained after oral administration. At the transcriptional level, castalagin downregulated genes involved in inflammatory pathways (NF-κB and AP-1) and cell migration (Rho GTPase). To the best of our knowledge, this is the first investigation in which ellagitannins from plant extracts have demonstrated a potential role in the interaction among *H. pylori* and human gastric epithelium.

## 1. Introduction

The discovery of *Helicobacter pylori* (*H. pylori*) infection by Warren and Marshall in 1983 is relatively recent, especially considering the subsequent efforts to convince the scientific community regarding its causal association with peptic ulcers, which led to the Nobel prize awarded in 2005 [1,2]. Gastric inflammation and oxidative stress are the clinical traits of acute and chronic gastritis, in which neutrophils, macrophages, and lymphocytes infiltrate the mucosa following chemokine attraction (for a comprehensive review, see Naito et al. [3]). The major inflammatory signal is the release of IL-8, whose levels correlate with *H. pylori* infection and gastric disease severity [4,5,6,7], but other mediators such as IL-6 and TNFα also play pivotal roles during disease progression [8]. Analysis of clinical biopsies revealed that activated NF-κB colocalizes with IL-8 in the *H. pylori*-infected mucosa of patients with gastritis [9], making the chemokine and NF-κB signaling key targets in the search for new therapies to treat *H. pylori*-induced gastritis and ulcers in humans. In the search for new therapies, the therapeutic approach to gastritis changed from mild diet interventions and prescription of antiacids to antibiotic therapy. Meanwhile, alternative approaches have been proposed to prevent or adjuvate the use of antibiotics, such as vaccines, probiotics, and food supplements [10,11].

Accordingly, whereas tannin-rich plants have been traditionally used against gastric diseases for long time, their direct anti-*H. pylori* effect has only been investigated during the last 20 years. To date, the hypothesis of potential benefits from tannin supplementation is mostly derived from rodent models of gastric ulcer (ethanol or FANS injury). The presence of ellagitannins in dietary products, such as fruits from *Punica granatum* L. and *Rubus* spp., contributes to their preclinical efficacy against gastric ulcer [12,13,14,15]. The antioxidant and anti-inflammatory properties of ellagitannins, observed through different experimental models [16,17], are supposed to contribute to the gastroprotective effect. Moreover, Funatogawa and colleagues suggested that ellagitannins also inhibit the growth of clinical strains of *H. pylori* at μM concentrations in microbiological tests [18]. Thus, the role of ellagitannins with reference to *H. pylori*-related gastritis still requires pharmacological validation.

*Castanea sativa* Mill. is a traditional source of polyphenols, mostly tannins. The chemical nature of chestnut tannins has recently been reported by several authors and our group; hydrolyzable tannins (ellagitannins and gallotannins) have been documented in bark [19,20], burs [21], flours [22], and leaves [23,24], while chestnut peel only contains condensed tannins [25]. The isomers castalagin and vescalagin were also identified as the main ellagitannins present in the bark [20]. Of note, other authors suggested that castalagin may counteract the inflammatory activity of human neutrophils and reduce the ethanol-induced ulcer formation in vivo [{Khennouf, 2003 #195}{Piwowarski, 2015 #234}], but its antibacterial and anti-inflammatory effect in *H. pylori* infection was poorly investigated.

Our previous work suggested the potential nutraceutical use in gastritis of chestnut peel from two botanical varieties (*Castanea sativa* Mill. var. venegon, and var. verdesa), collected from a specific area of Northern Italy (Campo dei Fiori, Varese) [25]. Similarly to chestnut peel, leaves may represent a sustainable plant material for pharmaceutical purposes in comparison to the bark. Of note, leaves have been traditionally used to treat gastrointestinal ailments, but the pharmacological properties and contribution of each component to the biological activity exerted by the extract still require a deep scientific assessment. 

The research on anti-gastritis compounds is affected by the challenge of creating reliable models of *H. pylori* infection, which naturally occurs in humans only [26,27,28]. For this reason, an in vitro model mimicking the human gastric epithelium–*H. pylori* interaction represents a useful tool for the evaluation of novel anti-gastritis candidates. 

In this work, we addressed the presence of polyphenols in *Castanea sativa* Mill. leaf extracts (var. venegon, and var. verdesa), with particular focus on ellagitannins, as a valuable polyphenol class for potential gastroprotective properties. Castalagin and vescalagin, commercially available as analytical standards, were identified and quantified in hydroalcoholic leaf extracts by LC–MS. Thus, we deeply investigated the anti-inflammatory and antibacterial activity of leaf extracts, in comparison with pure castalagin, an ellagitannin typically present in the leaves of *Castanea sativa* Mill., in a model of human gastric epithelium (GES-1) infected by *H. pylori*.

## 2. Materials and Methods

### 2.1. Materials

RPMI 1640 medium, penicillin, streptomycin, L-glutamine, and trypsin-EDTA were purchased from Gibco (Life Technologies Italia, Monza, Italy). Fetal bovine serum (FBS), and disposable materials for cell culture were purchased from Euroclone (Euroclone S.p.A., Pero-Milan, Italy). SV-40 immortalized GES-1 cells from human gastric epithelium were kindly donated by Dr. Dawit Kidane-Mulat, The University of Texas at Austin. Mueller–Hinton Broth, Brucella Broth, and glycerol from BD (BD, Franklin Lakes, NJ, USA), agar from Merck Life Science (Milan, Italy), and defibrinated sheep blood from Thermo Fischer Scientific (Oxoid^TM^ Hampshire, Basingstoke, UK) were used to cultivate and store *Helicobacter pylori*, cag+ strain 26695 from ATCC (ATCC 700392^TM^, Manassas, VA, USA). The reagents 3-(4,5-dimethylthiazol-2-yl)-2,5-diphenyltetrazolium bromide (MTT) and fluorescein-5-isothiocyanate (FITC) were purchased from Merck Life Science (Milan, Italy). Lipofectamine^®^ 3000 and carboxyfluorescein succinimidyl ester (CFSE) 5 mM (CellTrace^TM^, Cell Proliferation kits) and ActinRed^TM^ 555 ReadyProbes^TM^ reagent were from Invitrogen (Thermo Fisher Scientific, Waltham, MA, USA). Britelite^TM^ Plus reagent was from Perkin Elmer (Milano, Italy). The ellagitannins castalagin and vescalagin (certified purity, >95%) were from Phytolab GmbH & Co. KG (Vestenbergsgreuth, Germany). All reagents used for the biological assays were HPLC-grade. Human TNFα and human IL-8 ELISA Development Kits were from Peprotech Inc. (London, UK). All chromatographic solvents were HPLC-grade or LC–MS-grade for MS experiments. Acetonitrile, methanol, ethanol, formic acid, hydrochloric acid, vanillin, and iron sulfate were from Merck Life Science (Milan, Italy).

### 2.2. Extract Preparation and Phytochemical Characterization

Leaves from *Castanea sativa* Mill. var. venegon (VN) and *Castanea sativa* Mill. var. verdesa (VR) were collected at the end of the blossoming period, from the regional natural park “Campo dei Fiori” (from Castello Cabiaglio and Brinzio area, respectively for VN and VR, Varese, Italy). The plant material was naturally dried for 48 h at room temperature, and then extracted following previously reported methods [25]. In brief, 2.5 g of milled leaves were extracted twice with 50 mL of ethanol/water 50:50 (hydroalcoholic extract) for 4 and 16 h, respectively, at room temperature under dark conditions. Then, plant debris was removed using Supervelox filter paper; the extracts obtained were frozen at −80 °C overnight, lyophilized, and maintained at −20 °C. Before the biological activity evaluation, the extracts were dissolved in sterilized distilled water and DMSO (H_2_O:DMSO 50:50, 25 mg/mL), before being stored in aliquots at −20 °C.

### 2.3. LC–MS/MS Analysis

Ellagitannins castalagin and vescalagin were detected in the methanol solution of leaf extracts, and further quantified by UPLC–MS analysis using Exion LCTM AC System (AB Sciex, Foster City, CA, USA) with a Synergi 4 μm Hydro-RP 80 A LC Column 150 × 4.6 mm (Phenomenex, Torrance, CA, USA), coupled to a Triple Quad^TM^ 3500 system (AB Sciex, Foster City, CA, USA) with an ESI (−) source. The mobile-phase solvents consisted of water 0.1% formic acid (A) and methanol (B) set as 95% A/5% B (0–5 min), 100% B (5–8 min), and 95% A/5% B (8–15 min), with a flow rate of 0.8 mL/min.

### 2.4. Total Phenol Content Assay

Total polyphenol content was determined according to Folin–Ciocâlteu’s method, as reported by Singleton and Rossi [29]. Freeze-dried leaf extracts (1 mg) were solubilized in 1 mL of water. Aliquots of 300 μL from different samples were mixed in test tubes with 1.5 mL of Folin–Ciocâlteu’s reagent diluted 10 times and 1.2 mL of 7.5% (*w*/*v*) sodium carbonate. The absorbance was measured at 765 nm using a UV–Vis spectrophotometer (Victor^TM^ X3, Perkin Elmer, Walthman, MA, USA). Gallic acid was used as the reference standard for the calibration curve (0–30 µg/mL). Results were expressed as the weight of gallic acid equivalents (GA eq.) per weight of dry extract (% *w*/*w*).

### 2.5. Cell Culture

Human gastric epithelial (GES-1) cells were cultivated in RPMI 1640 medium, supplemented with penicillin 100 units/mL, streptomycin 100 mg/mL, L-glutamine 2 mM, and 10% heat-inactivated FBS. Cells were incubated at 37 °C, 5% CO_2_, in a humidified atmosphere. The subculture in new flasks and fresh medium (1 × 10^6^ cells) was repeated every 48–72 h upon reaching confluency, by detaching cells with trypsin ethylenediaminetetraacetic acid (EDTA) 0.25% solution. 

### 2.6. Bacterial Culture 

*H. pylori* cag+ strain 26695 was cultured in Petri dishes with Mueller–Hinton Broth medium, 5% agar, and 25% blood, for 72 h, under a microaerophilic atmosphere (5% O_2_, 10% CO_2_, and 85% N_2_) at 37 °C, 100% humidity. For the biological experiments, the bacterium was collected from the Petri dishes and counted according to its optical density at 600 nm (an OD value of 5 corresponds to 2 × 10^8^ bacteria). 

### 2.7. Cell Treatment

GES-1 cells were seeded in a 24-well plate (3 × 10^4^ cells/well) or 12-well plate (6 × 10^4^ cells/well), according to the specific investigation. Cells were treated with the proinflammatory stimulus TNFα (10 ng/mL) or *H. pylori* (bacterium-to-cell ratio of 50:1) for 1 h or 6 h, along with the leaf extracts (*Castanea sativa* Mill. var. verdesa and var. venegon) at different concentrations. The day before the bacterial infection, serum starvation was performed using 0.5% serum medium, supplemented with 1% L-glutamine and 1% penicillin/streptomycin. All the infection treatments were conducted with serum- and antibiotic-free medium, while TNFα treatments were conducted only with serum-free medium. During the treatment, cells were maintained in an incubator at 37 °C and 5% CO_2_. EGCG (50 µM) and procyanidin A2 (500 µM) were used as reference inhibitors of inflammatory markers and bacterial adhesion, respectively [11,30,31,32].

### 2.8. Cytotoxicity Assay

The integrity of the cell morphology before and after treatment was assessed by light microscope inspection. Cell viability was measured, after 6 h treatment (24-well plate, 3 × 10^4^ cells/well), using the 3,4,5-dimethylthiazol-2-yl-2-5-diphenyltetrazolium bromide (MTT) method [33]. This method evaluates the activity of a mitochondrial enzyme, which is an index of cell viability. Briefly, the medium was discarded; then, 200 μL of MTT solution (0.1 mg/mL, phosphate-buffered saline (PBS) 1×) was added to each well (45 min, 37 °C) and kept in darkness. Then, MTT solution was discarded, the remaining purple salt was dissolved in isopropanol/dimethyl sulfoxide (DMSO) (90:10 *v*/*v*), and the absorbance was read at 595 nm (Victor^TM^ X3, Perkin Elmer, Walthman, MA, USA). The absorbance of the solvent (blank) was subtracted from each value.

Ellagitannins and leaf extracts had no significant impact on cell viability up to the concentrations of 50 µM and 100 µg/mL, respectively (Figure S1). The impact on cell viability up to the concentrations used for 1 h treatments, i.e., 200 µM and 250 µg/mL, respectively, was also excluded.

### 2.9. Measurement of IL-8 Release

Cells were seeded at the density of 3 × 10^4^ cells/well (24-well plate) for 48 h. The chemokine IL-8 was quantified in cell medium in at least three independent experiments, after 6 h treatments with TNFα or *H. pylori* and the extracts, using a sandwich enzyme-linked immunosorbent assay (human IL-8 ABTS ELISA Development Kit, Peprotech). The assay was conducted according to manufacturer’s instructions, as previously reported [34]. The absorbance of the sample at 405 nm was compared to the absorbance of human recombinant standard IL-8 (0–1000 pg/mL). Data (mean ± SEM of at least three experiments) were expressed as the percentage relative to stimulated control, to which the value of 100% was arbitrarily assigned.

### 2.10. NF-κB Activation

The NF-κB pathway was evaluated by immunofluorescence and plasmid transfection, to measure the translocation of p65 subunit into cell nuclei or κB element-driven transcription, respectively, in at least three independent experiments.

#### 2.10.1. Immunofluorescence

The immunofluorescence technique was applied for the evaluation of NF-κB (p65 subunit) translocation into GES-1 nuclei; cells were challenged with TNFα or *H. pylori* and treated with the extracts, as previously described [34]. Briefly, cells were cultivated (3 × 10^4^/well) on coverslips placed in 24-well plates for 24 h. *H. pylori* was stained with CFSE 5 mM (2 µL of CFSE: 5 × 10^8^ bacteria) and incubated for 20 min at 37 °C; then, the bacterial suspension was supplemented with FBS, washed three times (PBS 1×), and centrifuged at 3150× *g* for 5 min to remove the excess of CFSE not bound to the bacterium. After 1 h treatment, the coverslips were washed (PBS 1×) and fixed with 4% formaldehyde solution for 15 min at r.t. A blocking solution (5% BSA) was added to the well and incubated at room temperature for 1 h. Cells were incubated with the primary antibody (NF-κB p65 (D14E12) XP^®^ Rabbit mAb #8242, Cell Signaling Technology, Danvers, MA, USA) diluted 1:400 *v/v* overnight at 4 °C, washed three times (PBS 1×), and then incubated with the secondary antibody (Alexa Fluor 647 conjugated with anti-rabbit immunoglobulin G (IgG) (heavy + light (H + L)), F(ab’)2 Fragment #4414, Cell Signaling Technology, Danvers, MA, USA) diluted 1:1000 *v*/*v*. A drop of ActinRed reagent, previously diluted 1:5 in PBS 1×, was added 30 min before the end of incubation. After 2 h, coverslips were washed with PBS and mounted on slides with a drop of ProLong Gold Antifade Reagent with 4′,6-diamidino-2-phenylindole (DAPI) (#8961, Cell Signaling Technology, Danvers, MA, USA), and then imaged using a confocal laser scanning microscope (LSM 900, Zeiss, Oberkochen, Germany).

#### 2.10.2. Measurement of the NF-κB-Driven Transcription

GES-1 cells were seeded in 24-well plates for 48 h (3 × 10^4^ cells), and then transiently transfected with a reporter plasmid, in which the luciferase gene was under the control of the E-selectin promoter containing three κB elements responsive to NF-κB (50 ng per well). Lipofectamine^®^ 3000 reagent was used for the transfection assays, according to the manufacturer’s instructions. The plasmid was a gift from Dr. N. Marx (Department of Internal Medicine-Cardiology, University of Ulm; Ulm, Germany). The day after, cells were treated with TNFα in addition to the extracts for 6 h. At the end of the treatment, the luciferase activity in cells was measured using Britelite^TM^ Plus reagent, according to the manufacturer’s instructions, as previously described [25]. Results (mean ± SEM of at least three experiments) were expressed as the percentage relative to the stimulated control, to which a value of 100% was arbitrarily assigned. 

### 2.11. Bacterial Adhesion to Cells

The bacterial adhesion was measured using a cytofluorimetric method adapted from Messing and colleagues [30]. GES-1 cells were seeded in 12-well plates for 48 h (6 × 10^4^ cells) before the infection with FITC-labeled *H. pylori*. FITC (2 μL) solution (1% in DMSO) was added to 10^8^ bacteria suspended in PBS 1× and incubated for 45 min at 37 °C; then, the bacterial suspension was centrifuged (3150× *g*, 5 min) and washed twice (PBS 1×) to remove the excess probe, before resuspending in PBS 1× for cell infection. Cell treatment (at least three independent experiments) was carried out for 1 h with extracts and a reference inhibitor (procyanidin A2, 500 μM), before (pretreatment) or after (cotreatment) infection. After 1 h (37 °C), cells were washed twice with PBS 1×, collected through a scraper in PBS/EDTA 2 mM, and centrifuged (3150× *g* for 5 min); then, they were fixed by formaldehyde (4% in PBS) and incubated in an ice bath for 10 min. Finally, cells were centrifuged, washed, and resuspended in 0.5% BSA (PBS/EDTA 2 mM) for analysis using the NovoCyte flow cytometer (ACEA Biosciences, San Diego, CA, USA) and NovoExpress software (ACEA Biosciences).

### 2.12. Minimum Inhibitory Concentration (MIC)

The microbroth dilution method was performed according to the recommendations of the Clinical and Laboratory Standards Institute (CLSI) [35] and was used to evaluate the MIC value, in at least three independent experiments. Extracts at different concentrations and the positive control (tetracycline 0.125 μg/mL) were prepared in 5% FBS Brucella broth; next, 100 μL of each sample were placed in a 96-well U-bottom plate. Then, 100 μL of *H. pylori* suspension (OD = 0.1) prepared in the same medium was added to each well. After mixing well, the 96-well plate was incubated at 37 °C in a 5% CO_2_ incubator under microaerophilic conditions. After 72 h, the rate of bacterial growth was measured using a microplate reader at 600 nm (Victor^TM^ X3, PerkinElmer, Waltham, MA, USA).

### 2.13. In Vitro Simulated Gastric Digestion

The gastric digestion was mimicked by an in vitro simulation, as previously described [34]. In brief, the extracts (100 mg) were incubated for 5 min at 37 °C with reconstituted saliva (6 mL); then, gastric juice (12 mL) was added, and the sample was incubated for 2 h at 37 °C under shaking. Finally, the suspension was centrifuged (5 min, 3000× *g*), and the supernatant was freeze-dried.

### 2.14. RNA Sequencing

GES-1 cells were seeded in a 12-well plate at a density of 6 × 10^4^ cells/well for 48 h and mRNA isolated using miRNeasy Mini Kit (Qiagen, Hilden, Germany). Cells were treated for 6 h with *H. pylori*, with or without castalagin (10 µM). Three replicates were used for each condition (ctrl, *H. pylori* treatment, *H. pylori*, and castalagin treatment). 

The quality and the concentration of the mRNA were assessed with RNA Screen Tape (Agilent Technologies, Santa Clara, CA, USA). The mRNA (500 ng) was fragmented and converted into complementary DNA (cDNA) through Illumina^®^ Stranded mRNA Prep (Illumina, San Diego, CA, USA) according to the manufacturer’s instructions. The quality and concentration of the final dual-indexed libraries were checked with D1000 Screen Tape (Agilent Technologies, Santa Clara, CA, USA). The NextSeq 500/550 High-Output Kit v2.5 was used to perform the sequencing through NextSeq 550 instrument (Illumina, San Diego, CA, USA).

### 2.15. RNA Sequencing Data Analysis 

For RNA sequencing data analysis, Illumina blc2fastq software was used to generate the Fastq files. The quality of each Fastq was inspected individually using the FASTQC tool vs. 0.11.9 [36], and Multiqc vs. 1.10.1 was then used to assess the overall good sequencing quality [37]. After the quality check, reads were aligned to the human reference genome GRCh38.p13 using STAR 2.7.9a [38], while Feature Counts 2.0.1 was used to obtain the counts for each sample [39] (see Table S1). The rlog function was applied to transform the count data, reducing differences among samples, and normalizing for the library size. Transformed data were used to perform principal component analysis (PCA) to evaluate the sample distribution and their clustering within groups representing the same condition. Normalization and differential gene expression analyses were carried out using the Bioconductor package DeSeq2 [40]. Following normalization, differentially expressed genes (DEGs) were detected. The Wald test in DESeq2 is the default test used for hypothesis testing. Genes were considered differentially expressed when they had AdjPval < 0.05 and log_2_FoldChange ≥ 0.58, indicating a fold change > 1.5 in either direction. The DEGs were then used to perform the enrichment analysis with the R package “EnrichR” [41]. The enriched pathways were determined using the GO_Biological_Process_2021 database.

### 2.16. Statistical Analysis

All biological results were expressed as the mean ± SEM of at least three independent experiments; the confidence interval related to the half-maximal inhibitory concentrations (IC_50_) calculation is reported in Section 3. Data were elaborated through an unpaired ANOVA test and Bonferroni post-hoc analysis. Statistical assessment and IC_50_ calculation were conducted using GraphPad Prism 8.0 software (GraphPad Software Inc., San Diego, CA, USA). Values of *p* < 0.05 were considered statistically significant.

## 3. Results

### 3.1. Phytochemical Characterization

*Castanea sativa* Mill. leaf is a well-known source of polyphenols, which include tannins and flavonoids [23,24]. According to our previous work on chestnut-derived extracts [25], we selected leaves from two varieties (*Castanea sativa* Mill. var. venegon and *Castanea sativa* Mill. var. verdesa) to perform a polar extraction with hydroalcoholic solvent (50:50 water/ethanol). The extraction yields were 16.24% and 21.88% (wt. of dry extract/wt. of dry plant material), respectively. 

According to the literature, tannins represent 6–8% *w*/*w* of polar extracts from leaves [23]. The chemical nature of tannins from leaves has been poorly investigated in comparison to the bark, which should contain high levels of hydrolyzable tannins (up to 10%), including ellagitannins, such as the isomers castalagin and vescalagin, and their hydrolysis products, castalin and vescalin [20]. For this reason, we firstly characterized the extracts from leaves using LC–MS analysis, thus confirming the presence of castalagin and vescalagin. 

The analytes, castalagin and vescalagin, were identified by comparing the retention time and the m/z with the respective standard compounds, using the following mass transitions: 933/631 (castalagin) and 933/613 (vescalagin) (Figure S2). The quantitative analysis was validated in terms of LOD and LOQ; thus, the ellagitannins were measured using the linear regression curve of the respective analytical standards (Table 1).

In parallel, we measured the total phenolic content in the extracts from venegon and verdesa varieties, which was 26.59% and 28.97% of weight GA eq./weight of dried extract, respectively (Table 2). The analysis was repeated after an in vitro simulated gastric digestion (see Section 2), and the process slightly diminished the total phenol level in both varieties, with statistically significant difference just for verdesa variety. 

Quantitative analysis using LC–MS/MS of castalagin and vescalagin showed that the two isomers overall represented 1.07% (10.69 μg/mg) and 1.24% (12.36 μg/mg) of the extracts from verdesa and venegon varieties, respectively. To gain more insight into the gastric stability of the two ellagitannins, their amount was also measured following the simulated gastric digestion as well. Reduction was statistically significant for both verdesa and venegon varieties: a fraction of castalagin (around 40% and 70%, respectively) was preserved, while vescalagin was not detectable after gastric digestion. The results are summarized in Table 3.

### 3.2. Castanea sativa Mill. Extracts Impair Inflammatory Markers Typically Increased during Gastritis

**Figure 1:**
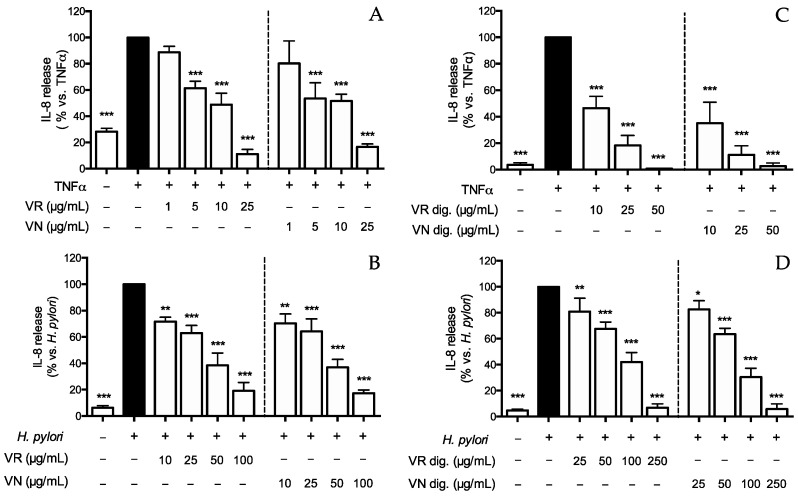
Effect of *Castanea sativa* Mill. leaf extracts on IL-8 release. GES-1 cells were treated for 6 h with TNFα (10 ng/mL) or *H. pylori* (ratio 50:1, bacteria/cell), in addition to leaf extracts before (**A**,**B**) and after in vitro simulated digestion (**C**,**D**). IL-8 was measured by ELISA assay. Results are expressed as the mean ± SEM (*n* = 3) of the relative percentage in comparison to stimulus (black bar), to which the value of 100% was arbitrarily assigned. * *p* < 0.05, ** *p* < 0.01, and *** *p* < 0.001 vs. stimulus. VR, *Castanea sativa* Mill. var. verdesa leaf extract; VN, *Castanea sativa* Mill. var. venegon leaf extract; dig., in vitro simulated digestion.

*H. pylori* infection and antibacterial effectors, such as TNFα, induce the release of IL-8 from gastric epithelial cells through the modulation of pathways converging in the NF-κB activation [6,42,43]. Thus, we carried out experiments to investigate the bioactivity of leaf extracts in both TNFα- and *H. pylori*-induced GES-1 cells (Figure 1). Firstly, we evaluated the effect of the leaf extracts on IL-8 release in cells challenged with TNFα (10 ng/mL): both the extracts inhibited IL-8 release in a concentration-dependent fashion (Figure 1A), with IC_50_ lower than 10 μg/mL. Then, the effect was also demonstrated on *H. pylori*-infected cells at slightly higher concentrations (IC_50_ < 30 μg/mL) (Figure 1B).

The in vitro simulated gastric digestion showed a moderate impact on the inhibitory activity (Figure 1C,D), as evident by the comparison of the IC_50_ values reported in Table 4.

**Figure 2:**
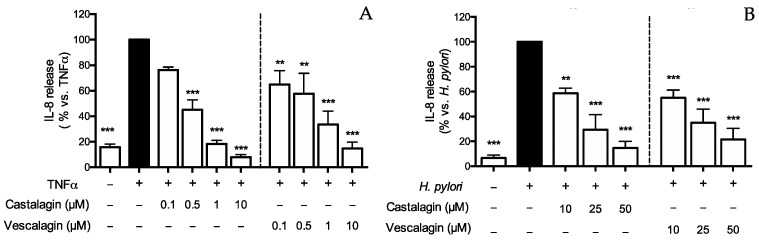
Effect of ellagitannins from *Castanea sativa* Mill. on IL-8 release. GES-1 cells were treated for 6 h with TNFα (10 ng/mL) (**A**) or *H. pylori* (ratio 50:1, bacteria:cell) (**B**), in the presence of castalagin or vescalagin. IL-8 was measured by ELISA assay. Results are expressed as the mean ± SEM (*n* = 3) of the relative percentage in comparison to stimulus (black bar), to which the value of 100% was arbitrarily assigned. ** *p* < 0.01, and *** *p* < 0.001 vs. stimulus.

Since castalagin and vescalagin were found in leaf extracts, and castalagin was partially stable after the simulated digestion, we addressed their potential effect on IL-8 secretion triggered by inflammatory stimuli or *H. pylori* infection. Ellagitannins strongly inhibited TNFα-induced IL-8 release with comparable IC_50_ (0.27 and 0.22 μM, respectively) (Figure 2A). Notably, they also inhibited *H. pylori*-induced IL-8 release, albeit at higher concentrations (Figure 2B). The IC_50_ values of castalagin and vescalagin with these parameters are summarized in Table 5.

Our group previously reported that ellagitannins can interfere with the NF-κB pathway in gastric epithelial models [25,44]. Consequently, we wondered whether the impairment of NF-κB pathway contributes to the IL-8 inhibition observed in the presence of the leaf’s extracts and pure ellagitannins. Firstly, we tested the underlying mechanism using the plasmid transfection assay, observing that extracts (25–100 μg/mL) and pure ellagitannins (0.5–10 μM) counteracted the NF-κB-driven transcription induced by TNFα (Figure S3). The bioactivity of the extracts was maintained after the simulated digestion, albeit at higher IC_50_ (>100 μg/mL), thus remarking that gastric environment may cause a partial reduction in chemical stability, in line with data regarding IL-8 release (Table 5). However, castalagin and vescalagin exhibited IC_50_ lower than 1 μM toward NF-κB-driven transcription, thus reflecting the inhibitory effect observed for leaf extracts (IC_50_ values are summarized in Table S2). 

**Figure 3:**
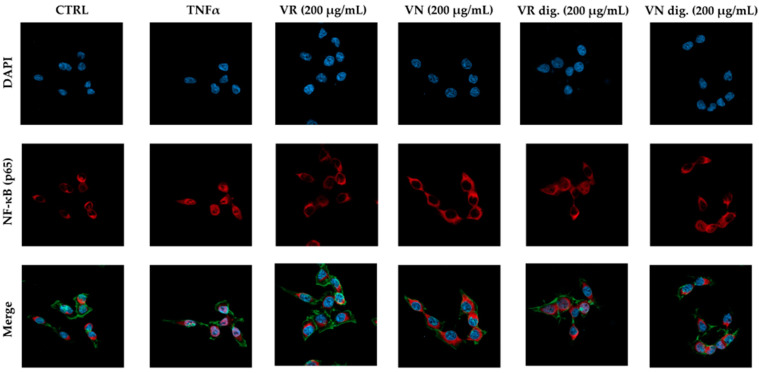
Effect of *Castanea sativa* Mill. leaf extracts on NF-κB (p65) nuclear translocation induced by TNFα. GES-1 cells were treated for 1 h with TNFα (10 ng/mL) in addition to leaf extracts (200 μg/mL) before and after in vitro simulated digestion. NF-κB (red) nuclear translocation from the cell cytoplasm into the nuclei (DAPI, blue) was detected by confocal immunofluorescence microscopy (63× objective). Merged images include β-actin staining (green). VR, *Castanea sativa* Mill. var. verdesa leaf extract; VN, *Castanea sativa* Mill. var. venegon leaf extract; dig., in vitro simulated digestion.

**Figure 4:**
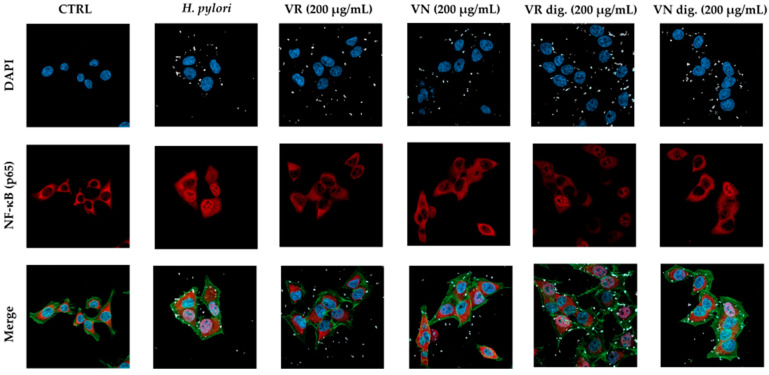
Effect of *Castanea sativa* Mill. leaf extracts on NF-κB (p65) nuclear translocation induced by *H. pylori*. GES-1 cells were treated for 1 h with *H. pylori* (ratio 50:1, bacteria/cell) in addition to leaf extracts (200 μg/mL) before and after in vitro simulated digestion. NF-κB (red) nuclear translocation from the cell cytoplasm into the nuclei (blue) was detected by confocal immunofluorescence microscopy (63× objective). Merged images include β-actin (green) and bacterial DNA (CFSE, white) staining. VR, *Castanea sativa* Mill. var. verdesa leaf extract; VN, *Castanea sativa* Mill. var. venegon leaf extract; dig., in vitro simulated digestion.

The impact on the NF-κB pathway was further evaluated by confocal microscopy experiments. At the selected concentration (200 μg/mL), the extracts interfered with NF-κB (subunit p65) translocation into the nucleus, during both TNFα (Figure 3) and *H. pylori* challenges (Figure 4). At the same concentration, the bioactivity of the extracts subjected to simulated gastric digestion was moderately affected, as previously observed.

**Figure 5:**
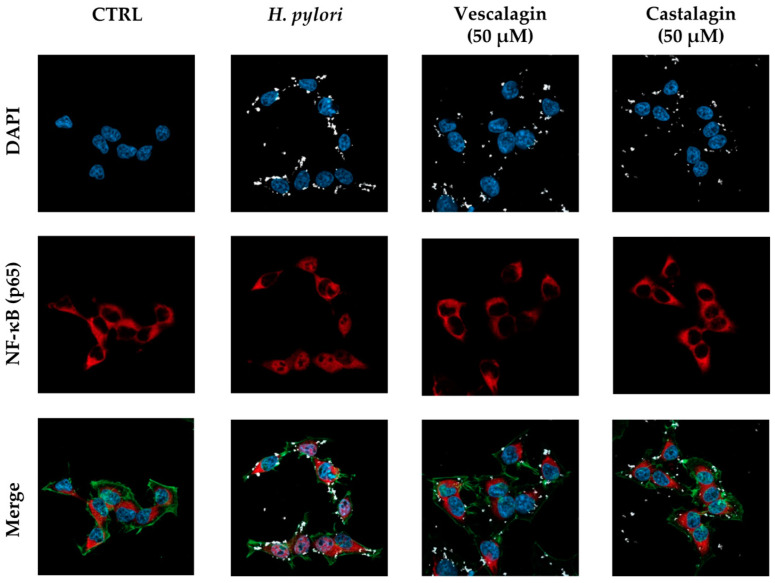
Effect of ellagitannins from *Castanea sativa* Mill. on NF-κB (p65) nuclear translocation induced by *H. pylori*. GES-1 cells were treated for 1 h with *H. pylori* (ratio 50:1, bacteria/cell) in addition to the ellagitannin isomers castalagin and vescalagin (50 μM). NF-κB (red) nuclear translocation from the cell cytoplasm into the nuclei (blue) was detected by confocal immunofluorescence microscopy (63× objective). Merged images include β-actin (green) and bacterial DNA (CFSE, white) staining. Cast., castalagin; Vesc., vescalagin.

In this case, we also confirmed the parallelism between the biological effects of the extracts and pure ellagitannins during *H. pylori* infection. As expected, castalagin and vescalagin impaired the nuclear translocation of p65 at the highest concentration responsible for the total inhibition of IL-8 release (50 μM) (Figure 5). The overall data sustain that the extracts may counteract IL-8 release by interfering with the NF-κB pathway, and that pure ellagitannins present in leaves may be responsible for this inhibitory mechanism. 

For this reason, we proceeded by comparing leaf extracts and ellagitannins for potential antibacterial effect against *H. pylori*, such as bacterial proliferation and adhesion to gastric cells.

### 3.3. Antibacterial Effect

A previous paper demonstrated the direct antibacterial effect of common monomeric ellagitannins on *H. pylori*, with MIC values below 25 μM [18]. However, to the best of our knowledge, castalagin and vescalagin have never been investigated for their antibacterial properties against *H. pylori*. Moreover, the antibacterial effect in coculture models of *H. pylori* infection has never been previously reported. For this reason, we decided to evaluate the impact of leaf extracts and their ellagitannins on bacterial growth and adhesion to gastric epithelial cells. 

**Figure 6:**
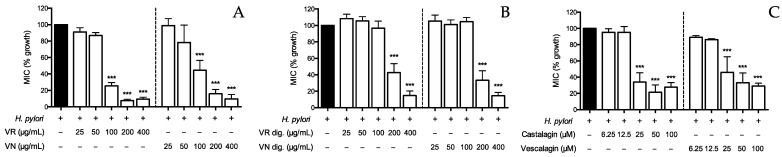
Effect of *Castanea sativa* Mill. leaf extracts and ellagitannins on *H. pylori* growth. *H. pylori* (OD = 0.1) was treated for 72 h with leaf extracts before (**A**) and after (**B**) in vitro simulated digestion, or ellagitannins (**C**). The rate of bacterial growth was measured as optical density (600 nm) using a photometer. Results are expressed as the mean ± SEM (*n* = 3) of the relative percentage in comparison to *H. pylori* growth (black bar), to which the value of 100% was arbitrarily assigned. *** *p* < 0.001 vs. *H. pylori*. MIC, minimum inhibitory concentration; VR, *Castanea sativa* Mill. var. verdesa leaf extract; VN, *Castanea sativa* Mill. var. venegon leaf extract; dig., in vitro simulated digestion.

Both the extracts impaired bacterial growth with MICs of 100 μg/mL, although the simulated digestive process caused a significant decrease in the bioactivity, increasing the MICs to 200 μg/mL (Figure 6A,B). Of note, castalagin and vescalagin showed MIC values of 25 μM (Figure 6C), similarly to other monomeric ellagitannins [18].

*H. pylori* adhesion to gastric epithelial cells is a crucial event for the pathogenesis of gastritis. Accordingly, we wondered whether leaf extracts and pure ellagitannins may counteract the bacterial adhesion through a direct effect on *H. pylori* or by acting on the adhesion machinery of gastric epithelial cells. Thus, we treated GES-1 cells for 1 h after (cotreatment) or before (pretreatment) *H. pylori* infection; the first experimental setting involved the interaction of the natural compounds with either the bacterium or human cell, whereas the second setting excluded the direct interaction with the bacteria, thus testing the involvement of variables related to the host cells.

**Figure 7:**
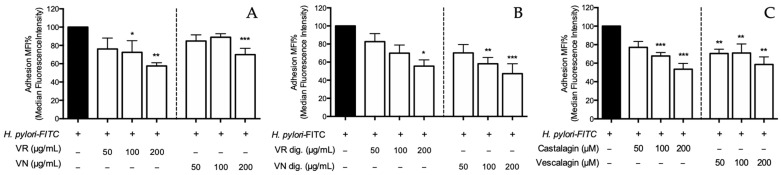
Effect of *Castanea sativa* Mill. leaf extracts and pure ellagitannins on *H. pylori* adhesion. GES-1 cells were treated for 1 h with *H. pylori*-FITC (ratio 50:1, bacteria/cell), in addition to leaf extracts before (**A**) and after (**B**) in vitro simulated digestion, or ellagitannins (**C**). The bacterial adhesion to GES-1 cells was measured as fluorescence intensity using a cytofluorimeter. Results re expressed as the mean ± SEM (*n* = 3) of the relative percentage in comparison to *H. pylori* infection (black bar), to which the value of 100% was arbitrarily assigned. * *p* < 0.05, ** *p* < 0.01, and *** *p* < 0.001 vs. *H. pylori*. MFI%, median fluorescence intensity; FITC, fluorescein isothiocyanate; VR, *Castanea sativa* Mill. var. verdesa leaf extract; VN, *Castanea sativa* Mill. var. venegon leaf extract; dig., in vitro simulated digestion.

Following the first experimental setting, the treatment with leaf extracts from verdesa and venegon varieties impaired the bacterial adhesion in a concentration-dependent fashion; the highest inhibitory effect was observed at 200 μg/mL (−42.5% and −30.1%, respectively), with comparable values after the simulation of gastric digestion (−44.5% and 52.8%, respectively) (Figure 7A,B). Castalagin and vescalagin showed an inhibitory activity within the concentration range of 50–200 μM (−46.5% and −41.4%, respectively, at the concentration of 200 μM) (Figure 7C). Of note, the inhibitory activity was observed at concentrations comparable to the MIC values, thus suggesting that antiadhesive properties may be due, at least in part, to the antibacterial activity.

**Figure 8:**
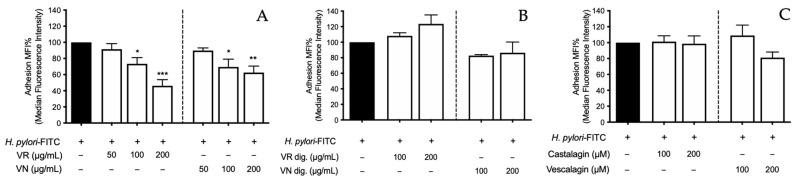
Preventive effect of *Castanea sativa* Mill. leaf extracts and ellagitannins on *H. pylori* adhesion. GES-1 cells were treated for 1 h with leaf extracts before (**A**) and after (**B**), or ellagitannins (**C**) in vitro simulated digestion, prior to *H. pylori*-FITC infection (ratio 50:1, bacteria/cell). The bacterial adhesion to GES-1 cells was measured as fluorescence intensity by cytofluorimeter. Results are expressed as the mean ± SEM (*n* = 3) of the relative percentage in comparison to *H. pylori* growth (black bar), to which the value of 100% was arbitrarily assigned. * *p* < 0.05, ** *p* < 0.01, and *** *p* < 0.001 vs. *H. pylori*. MFI%, median fluorescence intensity; FITC, fluorescein isothiocyanate; VR, *Castanea sativa* Mill. var. verdesa leaves extract; VN, *Castanea sativa* Mill. var. venegon leaves extract; dig., in vitro simulated digestion.

When experiments were carried out following the pretreatment setting, the extracts showed significant antiadhesive properties at 200 μg/mL (−53.8% and −27.5%, respectively) (Figure 8A), thus suggesting not only a direct effect on *H. pylori* during infection but also a potential impact on host cell variables. However, the simulated digestion abolished this effect (Figure 8B). Accordingly, castalagin and vescalagin were ineffective until the highest concentration tested (200 μM) (Figure 8C). This observation led us to hypothesize that direct antibacterial activity might represent the main explanation for the antiadhesive properties. However, it is not possible to exclude potential interference with the plethora of virulence factors involved during bacterial adhesion to host cells, as further mechanisms. This intriguing aspect is still poorly investigated referring to ellagitannins and related plant sources, thus demanding further host–pathogen interaction studies.

### 3.4. RNA-Seq Analysis

We then wondered whether the gastroprotective properties of ellagitannins may be partly due to or be mediated by changes in the epithelial cell transcriptome. Therefore, we carried out RNA-seq analysis of GES-1 cells infected with *H. pylori* without or upon treatment with castalagin (10 μM). 

The principal component analysis (Figure S4) showed that replicates clustered very well according to their condition (*H. pylori* vs. castalagin); in fact, the analysis showed a clear separation between replicates of infected cells and replicates of infected cells subjected to castalagin treatment. The normalized counts obtained through DESeq2 analysis are reported in the Table S1.

**Figure 9:**
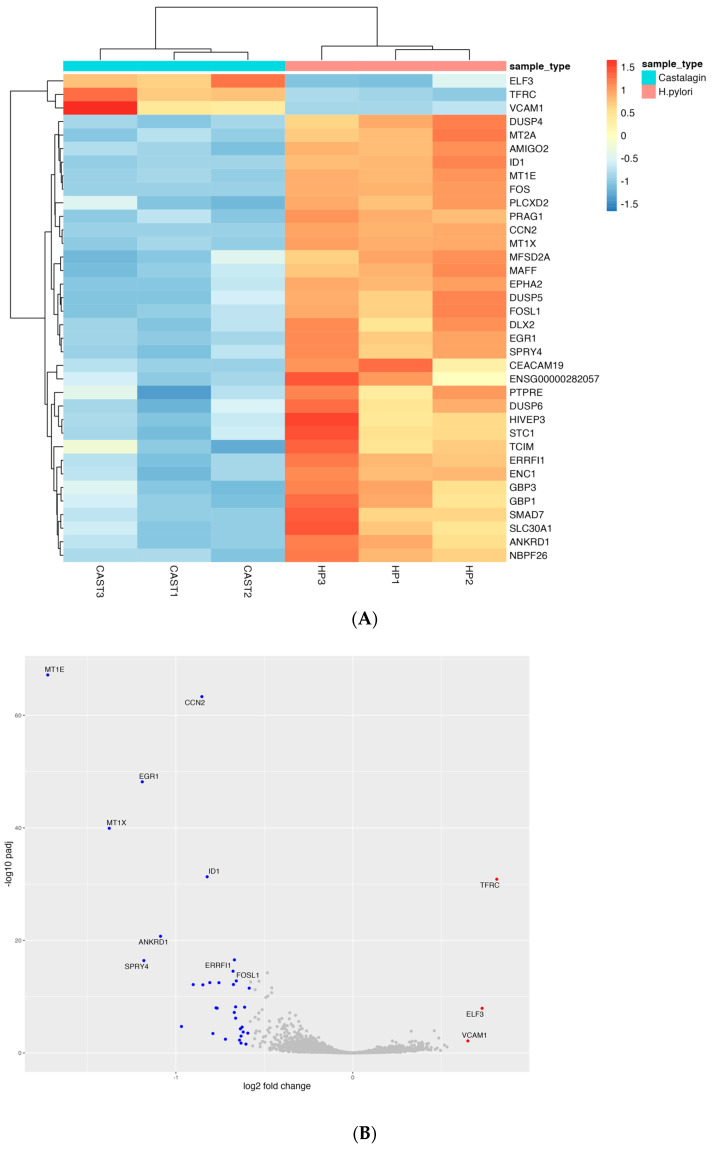
Heatmap showing the trend of the 36 differentially expressed genes between samples treated with *H. pylori* and samples treated with both *H. pylori* and castalagin (**A**). Volcano plot representing the downregulated and upregulated genes with blue and red dots, respectively (**B**).

A total of 36 differentially expressed genes were identified with three upregulated and 33 downregulated genes in RNA samples from *H. pylori*-infected cells treated with castalagin compared to RNA samples from infected cells with no other treatment (Figure 9A,B). 

Of note, considering the same subgroup of genes, most of them (29 out of 36) were regulated by *H. pylori* versus control uninfected cells, thus indicating that castalagin was able to specifically revert some of the transcriptional changes induced by *H. pylori* infection (Table 6). 

Lastly, the 33 downregulated genes were used to perform an enrichment analysis to evaluate which pathways were over-represented; the identified significant pathways with the corresponding number of genes are reported in Table 7. The enriched pathway analysis suggested that the main transcriptional targets of castalagin are as follows: signals involved in response to cytokines, including transcription factors (AP-1 and NF-κB) and MAPKs (ERK1/2), transport and signals related to divalent ions, and DNA-regulatory proteins, such as targets of the polymerase II (Figure S5). 

## 4. Discussion

*Castanea sativa* Mill. is a well-known source of polyphenols, including gallotannins and ellagitannins, with potential gastroprotective properties. It is remarkable that byproducts from chestnut harvesting, such as chestnut peel and leaves, represent a source of bioactive compounds with potential nutraceutical use. We previously reported that the outer tissues from chestnuts, belonging to var. venegon and var. verdesa (*Castanea sativa* Mill.), are rich in proanthocyanidins, showing anti-inflammatory effects on gastric epithelial cells [25]; however, chestnuts are devoid of ellagitannins, contrarily to what reported for the bark [19,20]. 

Ellagitannins have been reported to possess anti-ulcer and antibacterial properties [12,13,14,18], but their potential role in *H. pylori*-related gastritis is still poorly investigated. In this work, we demonstrated the presence of ellagitannin isomers, castalagin and vescalagin, in hydroalcoholic extracts from leaves belonging to both varieties from *Castanea sativa* Mill. We validated their pharmacological activity in a model of the nontumoral gastric epithelium (GES-1), challenged with TNFα or *H. pylori* infection.

Extracts from leaf and pure ellagitannins that are typically found in *Castanea sativa* Mill. inhibited the release of IL-8 from GES-1 cells, with more pronounced activity against TNFα challenge (Table 5 and Table 6). The mechanism of action may be ascribed, at least in part, to the impairment of the NF-κB pathway (Figure 3, Figure 4, Figure 5 and Figure S3), a known crossroad of TNFα- and *H. pylori*-induced molecular signaling [43]. However, it is plausible that other inflammatory targets were involved since, in our experiments, IL-8 release was always inhibited at lower concentrations than those impairing the NF-κB cascade. 

The same parallelism among leaf extracts and pure ellagitannins was observed in respect to the direct antibacterial and the antiadhesive properties against *H. pylori*. Our results are in line with those obtained by other authors, reporting that casuarinin, structurally similar to castalagin and vescalagin, could inhibit the growth of *H. pylori* at 13.35–26.70 μM [18]. Of note, the same authors suggested a higher specificity for *H. pylori* than *E. coli* (MIC > 100 μg/mL). Further investigations should clarify the antibacterial mechanism of action and the potential selectivity.

Regarding the possibility to attribute the bioactivity of leaf extracts to castalagin and vescalagin as main compounds, it is worth mentioning that the anti-inflammatory properties of the extracts showed a clear parallelism with the activities exhibited by ellagitannin isomers. The latter were selected as available compounds, representative for the class of ellagitannins, widely reported in *Castanea sativa* Mill. Indeed, especially during *H. pylori* challenge, pure ellagitannins exhibited their inhibitory effects at concentrations higher than those present in the extracts, thus suggesting the contribution of other hydrolyzable tannins, including ellagitannins, to the biological effect of the extract. 

To obtain a more comprehensive profile of the biological activities of ellegitannins, castalagin (10 μM) was selected as a candidate compound for RNA-seq experiments. This high-throughput approach allows capturing transcriptional changes and identifying target genes and pathways. A small set of genes (*n* = 36) was reverted by castalagin treatment with respect to *H. pylori* infection; most of them were downregulated (33), thus suggesting the prevalence of an inhibitory effect on transcription. Of note, a subset of downregulated genes (*tfrc*, *spry4*, *gbp1*, *gbp3*, *prag1*, *amigo2*, and *ptpre*) were related to the Rho GTPase pathway; among them, *amigo2* has been suggested as a biomarker of poor prognosis in gastric adenocarcinoma [45,46,47]. Despite the restricted number of modulated genes, PCA showed that the group of data related to castalagin treatment clustered from *H. pylori* infection, thus reflecting a significant difference among groups. 

The impact of ellagitannins on the Rho GTPase pathway is still poorly described, although other sources of ellagitannins have been investigated for their anti-inflammatory and antitumoral effect at the gut level [48]. Regarding inflammation, we could not exclude, on the basis of the literature concerning ellagitannins [16], that post-transcriptional and enzymatic mechanisms may have contributed to IL-8 inhibition in our setting.

Altogether, our data suggest that the use of the whole extract, accurately standardized for bioactive compounds such as castalagin and vescalagin, may exert more beneficial activity with respect to single compounds in gastritis. Other polyphenols, whose presence has been documented in *Castanea sativa* Mill. leaves, such as gallotannins or flavonol-derivatives [23,24], have previously been reported for anti-inflammatory and antibacterial properties at the gastric level [14,49]. In summary, our work attributed a relevant role to ellagitannins in the impairment of *H. pylori* viability and infection-induced inflammation in human gastric epithelial cells. 

## 5. Conclusions

Our work sustains, for the first time, the characterization of ellagitannins as bioactive principles in *Castanea sativa* Mill. leaf extracts. We suggest that leaves should be taken into consideration as suitable plant material to produce sustainable and bioactive extracts. Moreover, to the best of our knowledge, this is the first study in which the bioactivity of ellagitannins and related plant extracts has been validated in a model mimicking the gastric epithelium–*H. pylori* interaction.

## Figures and Tables

**Table 1:** Results of linear regression analysis of the LC-MS/MS.

Compound	Equation	R^2^	LOD (ng/μL)	LOQ (ng/μL)
Castalagin	y = 74284x − 727.79	0.9999	0.012	0.015
Vescalagin	y = 7929.8x − 46.85	0.9998	0.026	0.053

LOD, limit of detection; LOQ, limit of quantification.

**Table 2:** Total phenolic content (wt.% GA eq./wt.% d.e.) of extracts from *Castanea sativa* Mill. leaves.

	Before Gastric Digestion	After Gastric Digestion
Total Phenol Content	GA eq. (% *w*/*w*) ± SEM	GA eq. (% *w*/*w*) ± SEM
VR	28.97 ± 0.47	19.1 ± 0.81 ***
VN	26.59 ± 1.05	23.26 ± 1.28

VN, *Castanea sativa* Mill. var. venegon; VR, *Castanea sativa* Mill. var. verdesa; GA eq., gallic acid equivalents; d.e., dry extract. *** *p* < 0.001; *n* = 4.

**Table 3:** Characterization of ellagitannins from *Castanea sativa* Mill. leaf extracts.

	Before Gastric Digestion	After Gastric Digestion
Ellagitannins (Castalagin)	Mean ± SEM (μg/mg)	Mean ± SEM (μg/mg)
VR	5.79 ± 0.2	2.26 ± 0.04 ***
VN	5.88 ± 0.1	4.07 ± 0.08 ***
Ellagitannins (Vescalagin)	Mean ± SEM (μg/mg)	Mean ± SEM (μg/mg)
VR	4.90 ± 0.27	N.D. ***
VN	6.48 ± 0.27	N.D. ***

VN, *Castanea sativa* Mill. var. venegon; VR, *Castanea sativa* Mill. var. verdesa; GA eq., gallic acid equivalents; d.e., dry extract. *** *p* < 0.001, *n* = 3.

**Table 4:** IC_50_ (μg/mL) of the *Castanea sativa* Mill. extracts on IL-8 release.

	Before Gastric Digestion		After Gastric Digestion	
Extracts vs. TNFα	IC_50_ (μg/mL)	CI (95%)	IC_50_ (μg/mL)	CI (95%)
VR	6.81	4.64 to 9.99	9.31	6.51 to 13.33
VN	4.30	2.11 to 8.79	7.09	2.96 to 16.74
Extracts vs. *H. pylori*	IC_50_ (μg/mL)	CI (95%)	IC_50_ (μg/mL)	CI (95%)
VR	28.22	19.77 to 40.28	70.76	52.14 to 96.03
VN	27.76	18.14 to 42.48	60.57	49.94 to 73.47

IC_50_, 50% inhibitory concentration; CI (%), confidence interval; VN, *Castanea sativa* Mill. var. venegon; VR, *Castanea sativa* Mill. var. verdesa.

**Table 5:** IC_50_ (μM) of IL-8 release: ellagitannins from *Castanea sativa* Mill.

**Ellagitannins vs. TNFα**	**IC_50_ (μM)**	**CI (95%)**
Castalagin	0.27	0.20 to 0.38
Vescalagin	0.22	0.07 to 0.66
**Ellagitannins vs. *H. pylori***	**IC_50_ (μM)**	**CI (95%)**
Castalagin	11.70	7.65 to 17.89
Vescalagin	10.86	5.28 to 22.35

IC_50_: 50% inhibitory concentration; CI: confidence interval.

**Table 6:** Table showing the 36 DEGs from RNA-seq analysis. Genes are reported with both the Ensembl ID and Gene Symbol.

	*H. pylori* vs. Castalagin			*H. pylori* vs. CTRL	
Gene ID	Gene Symbol	Log_2_FoldChange	Padj	Log_2_FoldChange	Padj
ENSG00000169715	MT1E	−1.725	0.000	1.142	0.000
ENSG00000118523	CCN2	−0.853	0.000	1.069	0.000
ENSG00000120738	EGR1	−1.190	0.000	3.966	0.000
ENSG00000187193	MT1X	−1.377	0.000	0.777	0.000
ENSG00000125968	ID1	−0.823	0.000	No change	-
ENSG00000072274	TFRC	0.814	0.000	−0.682	0.000
ENSG00000148677	ANKRD1	−1.087	0.000	0.956	0.000
ENSG00000116285	ERRFI1	−0.669	0.000	2.540	0.000
ENSG00000187678	SPRY4	−1.181	0.000	2.585	0.000
ENSG00000175592	FOSL1	−0.677	0.000	3.425	0.000
ENSG00000142627	EPHA2	−0.659	0.000	1.805	0.000
ENSG00000120875	DUSP4	−0.808	0.000	1.559	0.000
ENSG00000115844	DLX2	−0.757	0.000	1.947	0.000
ENSG00000171617	ENC1	−0.675	0.000	0.699	0.000
ENSG00000117228	GBP1	−0.902	0.000	1.952	0.000
ENSG00000275342	PRAG1	−0.848	0.000	No change	-
ENSG00000185022	MAFF	−0.586	0.000	4.396	0.000
ENSG00000138166	DUSP5	−0.663	0.000	2.125	0.000
ENSG00000139211	AMIGO2	−0.612	0.000	0.892	0.000
ENSG00000125148	MT2A	−0.773	0.000	0.856	0.000
ENSG00000170345	FOS	−0.767	0.000	0.954	0.000
ENSG00000163435	ELF3	0.731	0.000	No change	-
ENSG00000101665	SMAD7	−0.670	0.000	No change	-
ENSG00000168389	MFSD2A	−0.663	0.000	2.679	0.000
ENSG00000170385	SLC30A1	−0.969	0.000	2.064	0.000
ENSG00000127124	HIVEP3	−0.626	0.000	1.717	0.000
ENSG00000176907	TCIM	−0.636	0.000	1.969	0.000
ENSG00000132334	PTPRE	−0.620	0.000	1.424	0.000
ENSG00000117226	GBP3	−0.593	0.000	1.082	0.000
ENSG00000139318	DUSP6	−0.791	0.000	No change	-
ENSG00000240891	PLCXD2	−0.632	0.001	0.796	0.000
ENSG00000159167	STC1	−0.720	0.004	1.767	0.000
ENSG00000282057	ENSG00000282057	−0.641	0.005	No change -	-
ENSG00000162692	VCAM1	0.650	0.007	2.183	0.000
ENSG00000186567	CEACAM19	−0.632	0.018	2.463	0.000
ENSG00000273136	NBPF26	−0.604	0.027	No change	-

**Table 7:** Table showing the identified enriched pathways using the 33 downregulated DEGs.

Term	Overlap	*p* Value	Padj	Genes
Cellular divalent inorganic cation homeostasis (GO:0072503)	5	1.01 × 10^−8^	8.93 × 10^−9^	MT2A; STC1; MT1X; SLC30A1; MT1E
Cellular response to cytokine stimulus (GO:0071345)	7	1.12 × 10^−9^	0.0005	EGR1; MT2A; ANKRD1; MT1X; FOS; GBP1; GBP3
Regulation of ERK1 and ERK2 cascade (GO:0070372)	5	4.14 × 10^−9^	0.001	DUSP4; CCN2; GBP1; DUSP6; EPHA2
Regulation of transcription, DNA-templated (GO:0006355)	10	0.002	0.025	FOSL1; EGR1; DLX2; TCIM; ID1; MAFF; ANKRD1; HIVEP3; FOS; SMAD7
Regulation of transcription by RNA polymerase II (GO:0006357)	9	0.008	0.055	FOSL1; EGR1; DLX2; ID1; MAFF; ANKRD1; HIVEP3; FOS; SMAD7
Positive regulation of transcription, DNA-templated (GO:0045893)	6	0.012	0.062	FOSL1; EGR1; MAFF; HIVEP3; FOS; SMAD7
Positive regulation of transcription by RNA polymerase II (GO:0045944)	5	0.012	0.066	FOSL1; EGR1; MAFF; FOS; SMAD7
Cellular protein modification process (GO:0006464)	5	0.025	0.081	DUSP4; DUSP5; PTPRE; PRAG1; DUSP6

## Data Availability

Data are available on request from the corresponding authors, E.D. and E.S. (emma.defabiani@unimi.it; enrico.sangiovanni@unimi.it).

## Supplementary Materials

The following supporting information can be downloaded at https://www.mdpi.com/article/10.3390/nu15061504/s1: Figure S1. Effect of *Castanea sativa* Mill. leaf extracts and ellagitannins on GES-1 viability (MTT test); Table S1. Total number of raw and normalized counts of the RNA-seq analysis for each sample; Figure S2. LC-MS chromatograms identifying the presence of castalagin and vescalagin in leaf extracts; Figure S3. Effect of *Castanea sativa* Mill. leaf extracts and ellagitannins on NF-κB driven transcription; Table S2. IC_50_ (μg/mL) of NF-κB driven transcription: extracts and ellagitannins from *Castanea sativa* Mill. leaves; Figure S4. Transcriptional effect of castalagin on *H. pylori*-induced gene expression in GES-1 cells. Enriched pathway analysis (A), volcano plot (B), and PCA (C); Figure S5. Summary of the relevant pathways according to P adjusted values (Padj).
