# Supplementary material for: Ellagitannins from Castanea sativa Mill. Leaf Extracts Impair H. pylori Viability and Infection-Induced Inflammation in Human Gastric Epithelial Cells
Source: Nutrients. 2023 Mar 21;15(6):1504. doi: 10.3390/nu15061504 (PMC10056456; doi:10.3390/nu15061504)
Supplement: Supplementary file 1 [file nutrients-15-01504-s001.zip › nutrients-2299488-supplementary.pdf]

Supplementary materials

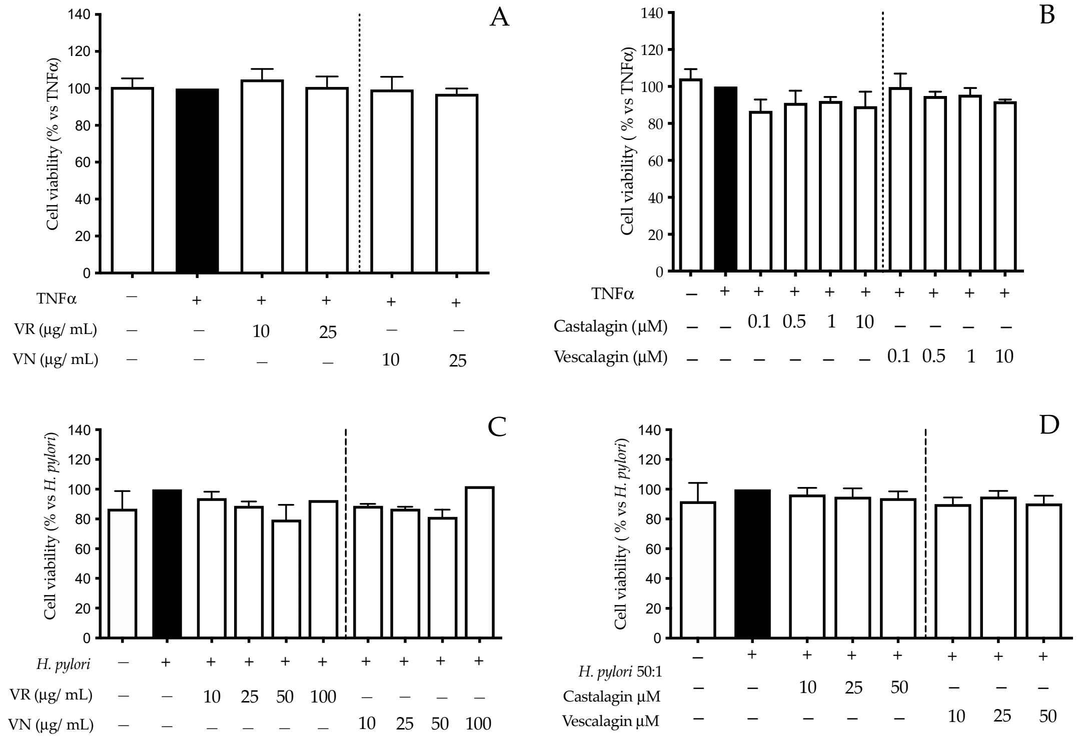

Figure S1. Cell viability (MTT test). GES-1 cells were treated for 6 h with TNF*α* (10 ng/mL) **(A, B)** or *H. pylori* (ratio 50:1, bacteria:cell) **(C, D)**, in addition to leaf extracts **(A, C)** or ellagitannins **(B, D)**. Results were expressed as mean ± SEM (n=3) of relative % in comparison to stimulus (black bar), to which the value of 100% was arbitrarily assigned. VR, *Castanea sativa* Mill. var. verdesa leaves extract; VN, *Castanea sativa* Mill. var. venegon leaves extract.

Table S1. Total number of raw and normalized counts of the RNA seq analysis for each sample, respectively.

Sample	Counts	Normalized Counts
Ctrl1	27361671	28054550
Ctrl2	28669496	27220474
Ctrl3	21053570	26461304
HP1	31452625	28978436
HP2	28707619	29315277
HP3	29898899	28793465
Cast1	28584314	28887177
Cast2	32328165	29108017
Cast3	42887810	28197446

Ctrl, control; HP, *H. pylori* treatment; Cast, *H. pylori* and castalagin treatment.

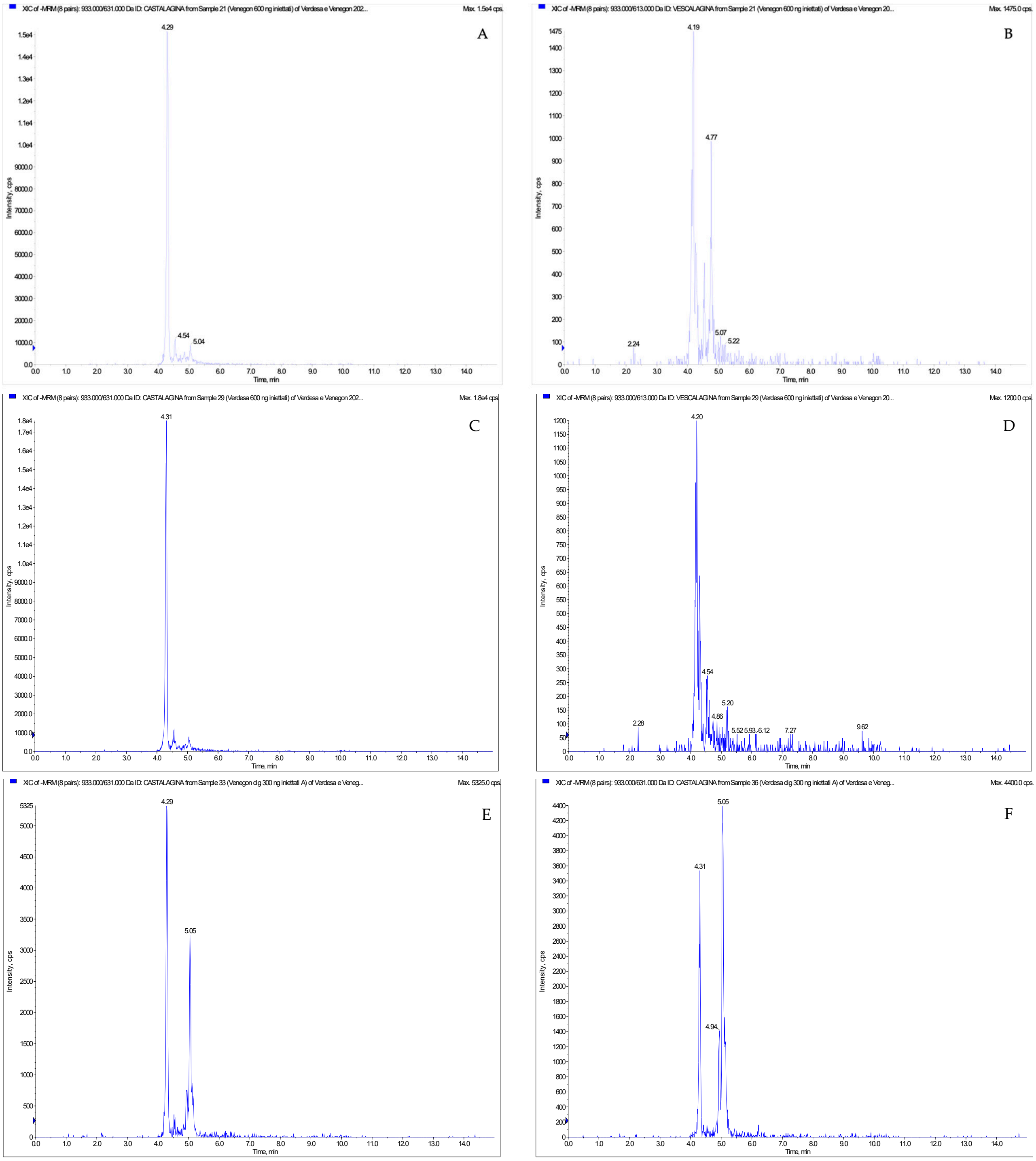

Figure S2. LC-MS chromatograms identifying the presence of castalagin and vescalagin in leaf extracts from *Castanea sativa* Mill. Castalagin **(A, C)** and vescalagin **(B, D)** were identified and quantified in the varieties venegon **(A, B)** and verdesa **(C, D)**. Castalagin was also detected in leaf extract from venegon (E) and verdesa (F) varieties after gastric simulated digestion.

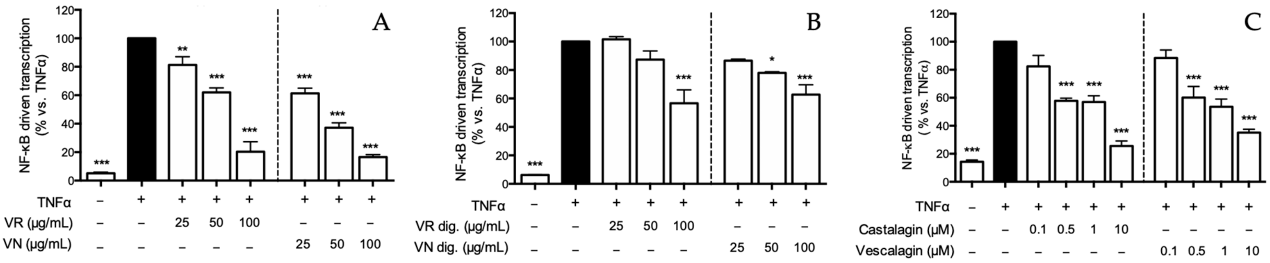

Figure S3. Effect of *Castanea sativa* Mill. leaves extracts and ellagitannins on NF-κB driven transcription. GES-1 cells were treated for 6h with TNFα (10 ng/mL), in addition to leaves extracts before **(A)** and after *in vitro* simulated digestion **(B)**, or ellagitannins **(C)**; NF-κB driven transcription was measured by plasmid transfection and luciferase assay. Results were expressed as relative % in comparison to TNFα (black bar), to which was arbitrarily assigned the value of 100%. *p<0.05, **p<0.01, ***p<0.001 vs TNFα. VR, *Castanea sativa* Mill. var. verdesa leaves extract; VN, *Castanea sativa* Mill. var. venegon leaves extract.

Table S2. IC_{50}s (μg/mL) of NF-κB driven transcription: extracts and ellagitannins from *Casteanea sativa* Mill. leaves.

**Extracts vs TNFα**	**IC_{50} (μg/mL)**	**I.C. (95%)**
VR	54.38	44.42 to 66.57
VN	31.87	27.68 to 36.68
**Digested extracts vs TNFα**	**IC_{50} (μg/mL)**	**I.C. (95%)**
VR	105.5	78.58 to 141.5
VN	151.5	86.68 to 264.7
**Ellagitannins vs TNFα**	**IC_{50} (μM)**	**I.C. (95%)**
Castalagin	0.71	0.4465 to 1.154
Vescalagin	0.83	0.4611 to 1.487

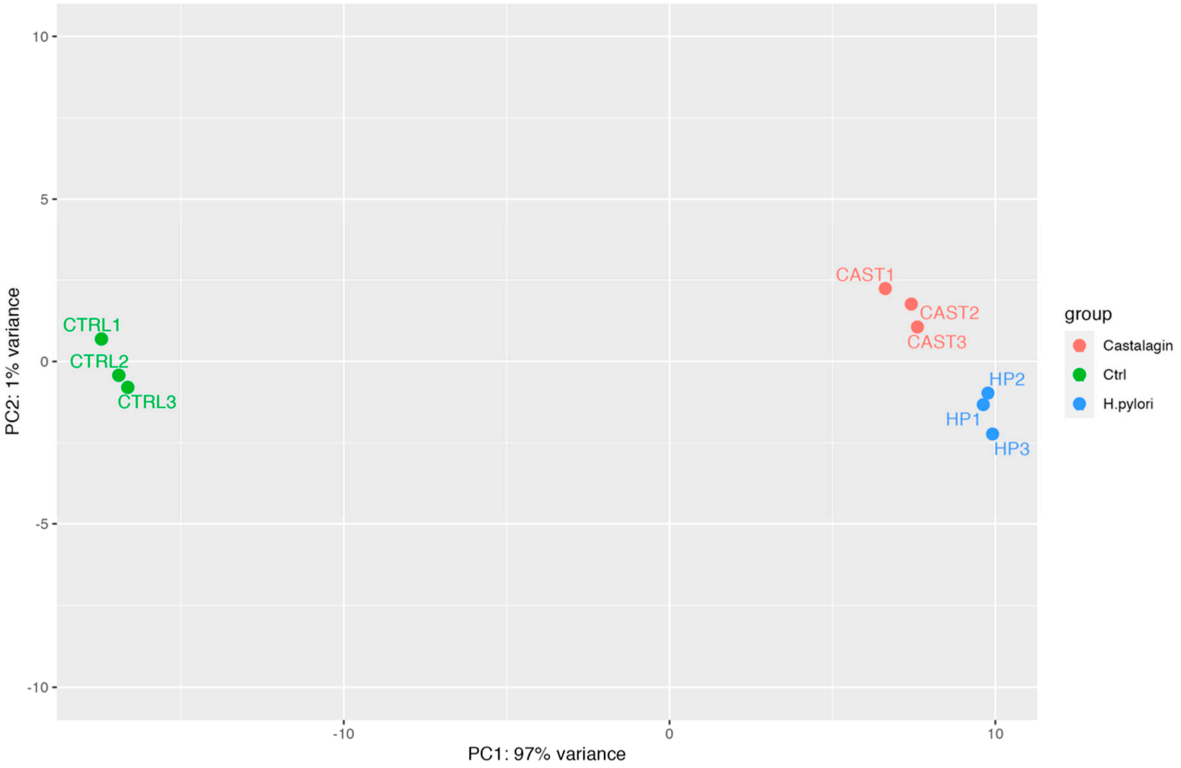

Figure S4. Transcriptional effect of castalagin on *H. pylori*-induced gene expression in GES-1 cells. GES-1 were treated for 6h with the ellagitannins castalagin (10 μM) and *H. pylori* (ratio 50:1, bacteria:cell) before RNA seq experiments. The PCA analysis for the clusterization of the experimental data is reported **(A)**; and enriched pathway analysis **(B)** of genes modulated by castalagin vs *H. pylori* are shown.

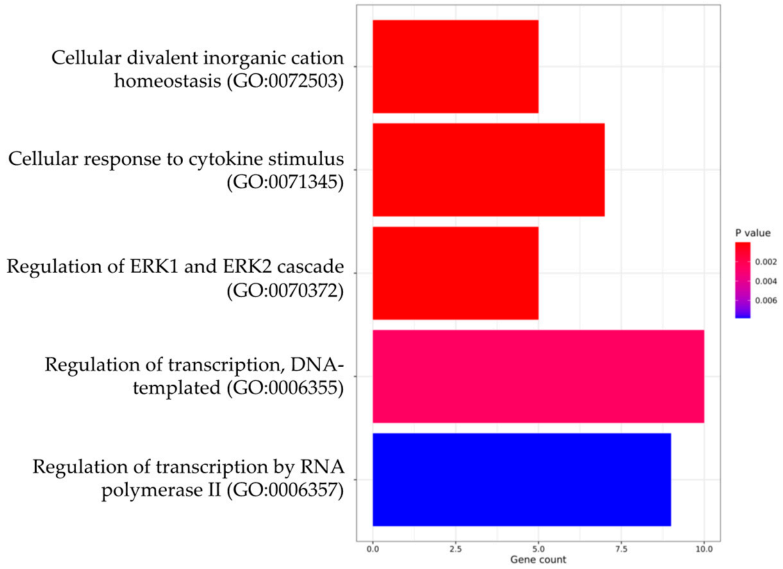

Figure S5. Summary of the relevant pathways according to P adjusted values (Padj).
